# No Gender Differences in Pain Perception and Medication after Lumbar Spine Sequestrectomy—A Reanalysis of a Randomized Controlled Clinical Trial

**DOI:** 10.3390/jcm11092333

**Published:** 2022-04-22

**Authors:** Christa K. Raak, Thomas Ostermann, Anna-Li Schönenberg-Tu, Oliver Fricke, David D. Martin, Sibylle Robens, Wolfram Scharbrodt

**Affiliations:** 1 Institute of Integrative Medicine, Witten/Herdecke University, 58313 Herdecke, Germany; o.fricke@gemeinschaftskrankenhaus.de (O.F.); david.martin@uni-wh.de (D.D.M.); w.scharbrodt@gemeinschaftskrankenhaus.de (W.S.); 2 Integrative Neuromedicine, Community Hospital Herdecke, Witten/Herdecke University, 58313 Herdecke, Germany; anna_tu@gmx.de; 3 Department of Psychology and Psychotherapy, Witten/Herdecke University, 58448 Witten, Germany; thomas.ostermann@uni-wh.de (T.O.); sibylle.robens@uni-wh.de (S.R.); 4 Department of Child and Adolescent Psychiatry, Psychotherapy and Child Neurology, Witten/Herdecke University, 58313 Herdecke, Germany

**Keywords:** gender differences, clinical trial, lumbar sequestrectomy, postoperative pain

## Abstract

Background: Gender issues have received increasing attention in clinical research of the past years, and biological sex has been introduced as a moderating variable in experimental pain perception. However, in clinical studies of acute pain and gender, there are conflicting results. In particular, there are limited data on the impact of gender differences after spinal sequestrectomy. The aim of this work is to examine gender differences in postoperative pain and pain medication consumption in an inpatient clinical setting. Methods: Data of a completed double-blind RCT was subdivided by gender and reanalyzed by means of an analysis of variance in repeated measures. Outcomes included pain severity measured on a VAS, affective (SES-A) and sensory pain perception (SES-S) and morphine equivalent doses (MED) of analgesics after spinal sequestrectomy. Results: In total, 42 female (47.73%) and 46 male (52.27%) patients were analyzed. No differences in pain severity (VAS: Gender × Time F = 0.35; (df = 2, 86); *p* = 0.708), affective and sensory pain perception (SES-A: Gender × Time F = 0.08; (df = 2, 86); *p* = 0.919; SES-S: Gender × Time F = 0.06; (df = 2, 86); *p* = 0.939) or post-operative opioid use between men and women (MEDs: Gender × Time F = 1.44; (df = 2, 86); *p* = 0.227) could be observed. Conclusions: This reanalysis of an RCT with respect to gender differences is to our knowledge the first attempt to investigate the role of gender in pain perception and medication after lumbar spine sequestrectomy. In contrast to other studies, we were not able to show significant differences between male and female patients in all pain-related outcomes. Apart from well-established pain management, psychological reasons such as gender-specific response biases or the observer effect might explain our results. Trial registration: The study was registered as a regulatory phase IV study at the German Clinical Trials Register (DRKS), an open-access online register for clinical trials conducted in Germany (Reg-No: DRKS00007913).

## 1. Introduction

According to recent statistics, the incidence of disc herniation is 5 to 20 cases per 1000 adults per year [1]. It is most common in people in their third to fifth decade of life, with a male-to-female ratio of 2:1. The approximate prevalence is about 1–3 percent of patients for symptomatic herniated discs of the lumbar spine [2]. In such cases, lumbar spine surgery is one of the most common procedures in the Western world. According to several studies and systematic reviews, there has been a rising trend in the total number of surgical interventions by 71% since 2007 in Germany [3].

Gender-specific perception of pain has been discussed frequently and has gained increasing attention in pain research in recent years [4]. Differences between women and men in pain appear to be related to both sex and gender. In abbreviated terms, the word “sex” refers to differences in human anatomy, physiology or organ systems, and the word “gender” refers to psychosocial interactions [5]. Most pain conditions have a higher prevalence in women, and women report more severe pain, longer pain duration and more frequent pain [6]. Studies have shown that female patients have higher pain intensity and require higher doses of opioids compared to male patients in the immediate postoperative period to achieve a similar level of analgesia [7]. In a brief review, Pieretti et al. examined literature on sex differences in experimental and clinical pain, focusing on biological mechanisms that have been suggested to be responsible for the observed sex differences. They found that biological factors such as sex hormones are considered to be one of the main mechanisms explaining differences in pain sensitivity in males and females [8].

Although gender differences in pain perception is a current research topic, little is known about its role in the field of degenerative diseases of the lumbar spine. Given these challenges, Maclean et al. [9] recently conducted a scoping review to map and synthesize the adult surgical literature regarding gender differences in pre- and postoperative patient-reported clinical assessment scores for patients diagnosed with lumbar degenerative disease. Postoperatively, female patients showed worse absolute pain, disability and quality of life, but showed equal or greater interval change compared with men [9]. Several clinical studies observed higher analgesic consumption after lumbar surgery in women than in men [10,11,12]. Most authors, however, conclude that further studies are needed to investigate gender differences in the effects of spine surgery.

Hence, we aimed at reanalyzing a recently conducted randomized, controlled clinical trial of patients undergoing elective, monosegmental, lumbar sequestrectomy [13] with respect to gender differences in pain perception, severity and pain medication use.

## 2. Materials and Methods

The original study was a regulatory, randomized controlled trial of phase IV, comparing additional treatment with potentized *Hypericum perforatum* to standard pain medication alone. The study was approved by the local ethics committee and the Federal Institute for Drugs and Medical Devices (BfArM, Bonn, Germany, EudraCT–No.: 2013-001383-31) [14].

### 2.1. Patients 

**Figure 1:**
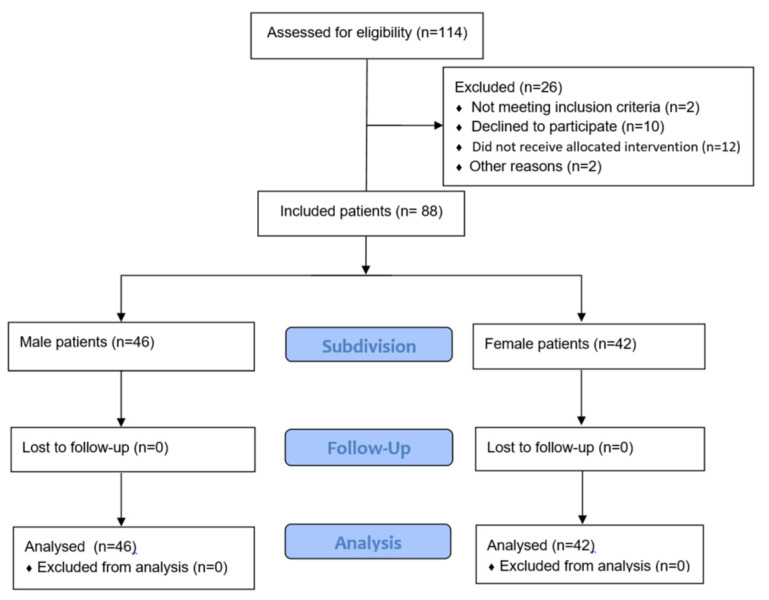
Flow chart of the patients included in the study.

A total of 114 study participants were recruited from November 2015 to August 2018 from patients receiving a monosegmental spinal sequestrectomy due to a lumbar disc herniation at the Department of Neurosurgery at the Community Hospital Herdecke. Of those, twelve patients were excluded and thus, a total of 88 patients were eligible for statistical analysis. Of those, two did not meeting inclusion criteria, ten declined to participate, twelve did not receive allocated intervention and two patients were excluded for other reasons. Thus, a total of 88 patients were included for statistical analysis. Figure 1 provides a flow chart of the patients included in the study.

### 2.2. Pain Medication and Outcomes

Patients were followed several times per day for their pain perception and medication use during their hospital stay. Standard pain medication included Ibuprofen and Metamizole, and in less frequent cases, Oxycodone, Tilidine or Tapentadol. If necessary, patients in few cases also received Morphine, Piritramide or Tramadol. Their number and dosages (mg) were extracted from the medical record folder and converted to morphine-equivalent doses (MEDs) in accordance with other trials on analgesic intake [13].

Pain perception was measured on a 100-mm visual analog scale (VAS) four times each day and then averaged for further evaluation. In addition, the German Version of the Pain Perception Scale introduced by Geissner was used to access the dimensions of “Affective Pain” (SES-A) and “Sensory Pain” (SES-S). The SES was scored at baseline and during postoperative study visits on days one, three and five [15]. 

### 2.3. Statistical Analysis

Baseline summary data of the total study population subdivided by gender were calculated using descriptive analyses and univariate statistics. To test for gender differences in pain management and perception, outcomes were modeled as a function of gender, duration of surgery and pain intensity at baseline within an ANOVA, including days after surgery as repeated measures (SAS-procedure PROC MIXED). A two-tailed error probability of α = 5% was used to test for gender differences. Results were reported using mean values and standard deviations for sample description and 95% confidence intervals for inferential statistics.

## 3. Results

In total, 42 female (47.73%) and 46 male (52.27%) patients were analyzed.

No significant differences were observed between the groups at baseline: female patients were aged between 25 and 82 years with a mean of 52.74 ± 12.85 years while male patients were aged between 18 and 79 years and on average 1.25 years younger (50.5 ± 14.42 years). Duration of surgery for all patients was about one hour (64.70 ± 24.73 min) without being significantly different between female (60.83 ± 23.35 min) and male (68.24 ± 25.66 min) patients. Body mass index also did not differ between females (26.93 ± 5.01) and males (28.02 ± 4.30). With respect to the indication of operation, an equal majority of the patients (*n* = 34, 38.6%) were diagnosed with a herniated disc at lumbar segments L5–S1 and L4–L5, followed by L3–L4 in 14 cases (15.9%), L2–L3 in 5 cases (5.7%) and in one case, L1–L2 (1.1%). As shown in Table 1, there was no significant difference in the distribution of affected lumbar segments between male and female patients. Also, the affected side did not significantly differ between male and female patients (*p* = 0.741): in 15 female (35.7%) and 18 male patients (39.1%) the right side was affected, while the left side was affected in 27 female patients (64.3%) and 28 male patients (60.9%). In two cases (one female, one male) the location according to the classification given in [16] was exclusively intraforaminal, while in one male patient, the location was intra-extraforaminal. Table 1 summarizes the sociodemographic data, anatomical location and surgical duration.

### 3.1. Pain Severity 

**Figure 2:**
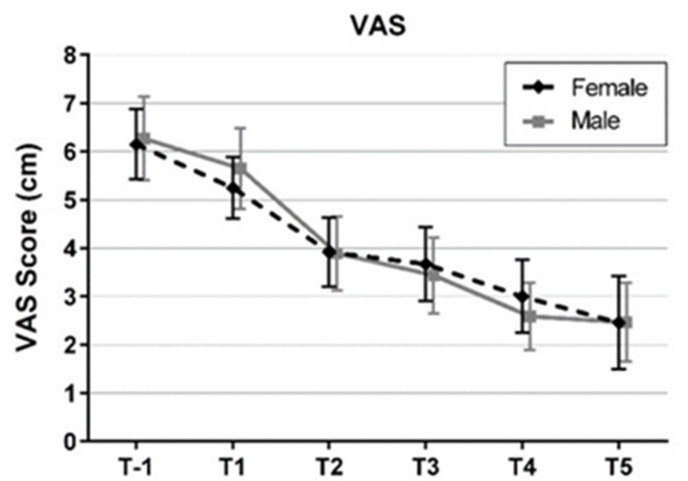
Means and 95% confidence intervals of pain severity from hospital admission (T-1) to day 5 (T5) by gender (VAS: visual analogue scale).

Figure 2 shows the development of the pain severity as measured with a VAS over the entire inpatient period. Regardless of gender, a clear decrease in pain perception by about 60% from 6.21 ± 2.59 at hospital admission to 2.46 ± 2.52 at day 5 was observed without being significantly different between gender in the complete course of time (ANOVA: Gender × Time F = 0.35; (df = 2, 86); *p* = 0.708) and for each of the single time points (*p* between 0.412 and 0.983). 

### 3.2. Affective and Sensory Pain Perception

Data on affective (SES-A) and sensory pain perception (SES-S) are provided in Table 2 and Table 3. 

In both groups there was a significant reduction in sensory pain perception, which resulted in almost identical values for affective pain perception (22.53 ± 11.15 in female patients and 23.11 ± 10.62 in male patients) and sensory pain perception (15.32 ± 5.53 in female patients and 15.33 ± 7.02 in male patients) on day five. Again, the linear mixed model did not reveal any significant differences in the course of time between the groups (ANOVA SES-A: Gender × Time F = 0.08; (df = 2, 86); *p* = 0.919; SES-S: Gender × Time F = 0.06; (df = 2, 86); *p* = 0.939).

### 3.3. Pain Medication

**Figure 3:**
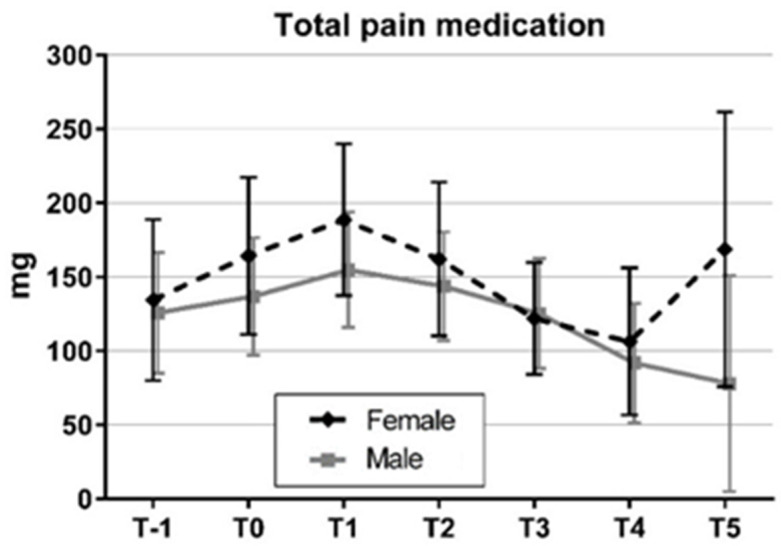
Means and 95% confidence intervals of total pain medication from hospital admission (T-1) to day 5 (T5) by gender.

Pain medication measured in MEDs increased from 129.94 ± 155.46 mg MED at admission to 149.97 ± 151.95 mg MED on day one and a maximum of 171.02 ± 148.16 mg MED on day 2 for all patients. Subdivided by gender pain medication showed an almost identical course in women and men, however, MEDs in men except for day three were always below the MEDs of the female patients (Figure 3) but without being significant (ANOVA: Gender × Time F = 1.44; (df = 2, 86); *p* = 0.227).

This is also reflected in the statistical analysis of the total amount of medication in the inpatient period subdivided by gender. Although there is a lower amount of medication in men, the difference is not significant (*p* = 0.47; Table 4).

## 4. Discussion

This reanalysis of an RCT with respect to gender differences is to our knowledge the first attempt to investigate the role of gender in pain perception and medication after lumbar spine sequestrectomy. Clinical and anatomical data of our study i.e., data on the level of disc herniation, side and location is in accordance with published data provided in [16,17,18]. No significant differences were observed between the groups at baseline in terms of age, pain medication, duration of operation and body mass index, which, in terms of preoperative opioid use, is in accordance with [19]. We were able to show that there were no differences between male and female patients for all pain-related outcomes. Remarkably, pain medication consumption showed an almost identical course in women and men, however, MEDs in men except for day three were always below the MEDs of the female patients but without being significant, in agreement with the results of [20]. The same is seen in the total amount of medication consumption: a lower amount of medication in men, but the difference is not significant, which contrasts with the results of [21] but is in line with the results of [22]. 

As mentioned in the introduction, most studies examining pain and gender differences find worse outcomes for women. Studies have shown this effect in both pre- and postoperative acute pain settings [8,23], which was also shown by a number of studies from the field of gender differences in surgical management of lumbar degenerative disease [9,24]. In another study of Strömquist et al. (2016) on preoperative data from 15,631 patients who underwent lumbar disc herniation surgery between 2000 and 2010, women were reported to have worse clinical status than men [11]. The study, however, found no evidence-based data to support this difference, and the reason for this finding remained unclear. Part of the explanation for the differences between men and women could be physical constitution, which leads to different biomechanical properties [25].

Sex hormones are often listed as influencing factors for gender differences, in addition to endogenous opioid activation, neurochemical mechanisms or differences in neuroimmunology and genetic factors matter [26,27]. Possible biopsychosocial and psychosocial factor mechanisms underlying sex differences in pain need to be discussed.

The prevalence of chronic low back pain is higher in women than men and increases linearly from the third decade of life to age 60 [28]. Therefore, women are more likely to experience clinical pain symptoms, and they show increased pain sensitivity in experimental pain studies [29].

Finally, these findings may simply reflect gender-specific response biases. Men underreport pain and/or women overreport pain. Humans of different genders also differ in pain management strategies. Male patients tend to prefer problem-solving and instrumental strategies, whereas female patients are more likely to seek social support and tend to focus more on emotional aspects of the pain situation [29,30]. In addition, depression and anxiety are also often associated with physical pain, with a higher prevalence in women [31].

A systematic literature review found that a so-called observer effect often influences studies: female study participants tended to decrease pain when the investigator was of the opposite sex, while men tended to rate pain lower when the investigator was female [32]. In our study, the outcomes were directly documented in the documentation sheets from the patients themselves, so this effect will not have played a role.

## 5. Limitations

The sample size of the study was based on an efficacy trial investigating the treatment with homeopathic *Hypericum perforatum* as an add-on to standard postoperative pain management. Thus, the trial was not intended to detect small differences such as those in the present reanalysis with regard to gender. However, statistical results with *p*-values clearly above the threshold of significance do not suggest that the low sample size plays an important role. 

All patients had been randomly assigned to receive either a placebo or *Hypericum perforatum*; blinding of the patients was carried out properly, and no gender differences in the original study outcomes were observed. Thus, a contamination of the present results due to positive expectancy regarding the therapy, which might have influenced pain perception, can also be ruled out. Moreover, in the original trial, the additional treatment with *Hypericum perforatum* did not show a significant effect with respect to pain perception or opioid consumption. Thus, an influence of the additional treatment with *Hypericum perforatum* can also be excluded [13].

## 6. Conclusions

The results of this reanalysis are contradictory to most other studies in the field of neurosurgery, as no significant gender differences in pain perception and analgesic consumption after lumbar spinal surgery could be presented. It is possible that biopsychosocial mechanisms and the role of psychological factors that may influence sex differences in pain do not occur during a short postoperative inpatient stay. This should be considered in future research. Moreover, a summary of existing findings, e.g., in terms of a systematic review or meta-analysis, would therefore be desirable.

## Figures and Tables

**Table 1:** Sociodemographics subdivided by gender.

	Total *n* = 88	Female *n* = 42	Male *n* = 46	*p*-Value
**Age (years)**				
*n*	88	42	46	
M ± SD	51.57 ± 13.66	52.74 ± 12.85	50.5 ± 14.42	0.446
Median	53	53.5	53	
Minimum	18	25	18	
Maximum	82	82	79	
**Surgery duration**				
*n*	88	42	46	
M ± SD	64.70 ± 24.73	60.83 ± 23.35	68.24 ± 25.66	0.162
Median	60	57.5	65	
Minimum	26	26	33	
Maximum	158	158	133	
**BMI**				
*n*	86	42	44	
M ± SD	27.48 ± 4.67	26.93 ± 5.01	28.02 ± 4.30	0.283
Median	27.05	25.31	28.54	
Minimum	19.13	19.13	19.15	
Maximum	40.09	40.09	38.58	
**Level of disc herniation**				
*n*	88	42	46	
L1–L2	1 (1.1%)	1 (2.4%)	0 (0.0%)	
L2–L3	5 (5.7%)	2 (4.8%)	3 (6.5%)	
L3–L4	14 (15.9%)	7 (16.7%)	7 (15.2%)	0.828
L4–L5	34 (38.6%)	17 (40.5%)	17 (37.0%)	
L5–S1	34 (38.6%)	15 (35.7%)	19 (41.3%)	
**Side**				
*n*	88	42	46	
right	33 (37.5%)	15 (35.7%)	18 (39.1%)	0.741
left	55 (62.5%)	27 (64.3%)	28 (60.9%)	
**Location**				
*n*	88	42	46	
exclusively intraforaminal	2 (2.3%)	1 (2.4%)	1 (2.2%)	
intra-extraforaminal	1 (1.1%)	0 (0.0%)	1 (2.2%)	0.629
intraspinal	85 (96.6%)	41 (97.6%)	44 (95.6%)	

**Table 2:** Affective pain perception (SES-A) from hospital admission (T-1) to day 5 after operation (T5). *p*-values of *t*-test comparisons between men and women.

SES-A		Total *n* = 88	Female *n* = 42	Male *n* = 46	*t*-Test *p*-Value
**T-1**	*n*	88	42	46	
	M ± SD	35.60 ± 11.50	36.71 ± 12.32	34.59 ± 10.74	0.389
	Median	36.00	40.00	35.50	
**T1**	*n*	88	42	46	
	M ± SD	24.30 ± 10.43	24.14 ± 10.24	24.43 ± 10.71	0.897
	Median	20.00	20.00	20.50	
**T3**	*n*	87	42	45	
	M ± SD	20.55 ± 8.61	20.14 ± 9.38	20.93 ± 7.92	0.671
	Median	17.00	16.50	18.00	
**T5**	*n*	37	19	18	
	M ± SD	22.81 ± 10.75	22.53 ± 11.15	23.11 ± 10.62	0.871
	Median	18.00	17.00	18.50	

**Table 3:** Sensory pain perception (SES-S) from hospital admission (T-1) to day 5 after operation (T5). *p*-values of *t*-test comparisons between men and women.

SES-S		Total *n* = 88	Female *n* = 42	Male *n* = 46	*t*-Test *p*-Value
**T-1**	*n*	87	41	46	
	M ± SD	21.10 ± 7.55	21.29 ± 7.35	20.93 ± 7.80	0.827
	Median	20.00	20.00	19.50	
**T1**	*n*	86	41	45	
	M ± SD	16.51 ± 6.70	16.63 ± 6.59	16.40 ± 6.88	0.873
	Median	14.50	14.00	15.00	
**T3**	*n*	86	42	44	
	M ± SD	14.06 ± 5.23	14.00 ± 5.06	14.11 ± 5.44	0.920
	Median	12.00	12.00	12.00	
**T5**	*n*	37	19	18	
	M ± SD	15.32 ± 6.21	15.32 ± 5.53	15.33 ± 7.02	0.993
	Median	13.00	14.00	13.00	

**Table 4:** Total amount of medication in mg MED from day 1 to day 5 after operation by gender.

	Total *n* = 88	Female *n* = 42	Male *n* = 46	*t*-Test
**Sum of day 1 to day 5**	Mean (SD)	Mean (SD)	Mean (SD)	*p*-value
Medication	569.57 (543.78)	614.09 (596.95)	528.92 (498.42)	0.47

## Data Availability

The datasets used and/or analyzed during the current study are available from the corresponding author upon reasonable request.
